# Auxin Response Factor Genes Repertoire in Mulberry: Identification, and Structural, Functional and Evolutionary Analyses

**DOI:** 10.3390/genes8090202

**Published:** 2017-08-25

**Authors:** Vinay Kumar Baranwal, Nisha Negi, Paramjit Khurana

**Affiliations:** Department of Plant Molecular Biology, University of Delhi South Campus, Benito Juarez Road, New Delhi 110021, India; vinaydu@gmail.com (V.K.B.); komalngi28@gmail.com (N.N.)

**Keywords:** auxins, ARFs, evolution, expression analysis, gene family, *Morus notabilis*

## Abstract

Auxin Response Factors (ARFs) are at the core of the regulation mechanism for auxin-mediated responses, along with AUX/IAA proteins.They are critical in the auxin-mediated control of various biological responses including development and stress. A wild mulberry species genome has been sequenced and offers an opportunity to investigate this important gene family. A total of 17 ARFs have been identified from mulberry (*Morus notabilis*) which show a wide range of expression patterns. Of these 17 ARFs, 15 have strong acidic isoelectric point (pI) values and a molecular mass ranging from 52 kDa to 101 kDa. The putative promoters of these ARFs harbour *cis* motifs related to light-dependent responses, various stress responses and hormone regulations suggestive of their multifactorial regulation. The gene ontology terms for ARFs indicate their role in flower development, stress, root morphology and other such development and stress mitigation related activities. Conserved motif analysis showed the presence of all typical domains in all but four members that lack the PB1 domain and thus represent truncated ARFs. Expression analysis of these ARFs suggests their preferential expression in tissues ranging from leaf, root, winter bud, bark and male flowers. These ARFs showed differential expression in the leaf tissue of *M. notabilis*, *Morus laevigata* and *Morus serrata*. Insights gained from this analysis have implications in mulberry improvement programs.

## 1. Introduction

Auxin—a phytohormone—has been shown to regulate many aspects of plant development, especially in apical dominance, root initiation, shoot elongation and shaping the pattern of embryos, etc. It is well known that the auxin/indole-3-acetic acid repressors (AUX/IAA), under low auxin concentrations, forms dimers with transcription factors of the auxin response factor (ARF) class [1]. After perceiving the signal, ubiquitin mediated degradation of Aux/IAA releases these ARFs and thus regulates the underlying genes by binding to the promoters with Auxin Responsive Elements (AREs). To date, several downstream genes have been identified including SMALL AUXIN UP RNA (*SAUR*), Gretchen Hagen 3 (*GH3*) and the indole-3-acetic acid-inducible gene (*Aux/IAA*) [2]. Structural analysis of these ARFs has revealed three conserved features including an N-terminal DNA-binding domain, a less conserved transcriptional regulation domain and the C-terminal protein interaction domain are involved in hetero or oligomerization. The DNA binding domain binds to ARE elements directly by virtue of its structural topology, while the protein interaction domain at the C-terminal leads either to homodimerization or binding to other AUX/IAA, thus facilitating the transcriptional block of these ARFs [3,4]. Several truncated ARFs that lack C-terminal motifs have also been reported [5]. Genome wide evolution, function and expression analyses in all major lineages have been conducted to delineate the ARF coding genes. Useful insights like the number, structure, phylogeny and functional niche have been gained from these analyses leading to a better understanding of auxin-mediated responses in higher plants. To date, in dicot systems, 23 ARFs from *Arabidopsis thaliana* [6], 39 ARFs from tree species *Populus trichocarpa* [7], 17 from *Eucalyptus grandis* [8] and 22 ARFs from tomato [9] etc., have been studied. In monocots, ARF components have been studied in detail in rice [10], *Sorghum* [11] and maize [12] systems. These studies revealed a wide range of roles for these transcription factors including stress response, fruit development, root and shoot morphology and embryo developments. The expression patterns of these ARFs have been shown to modulate in responses like abscission and seed development. Furthermore, ARFs have been shown to be important for linking hormonal crosstalk of auxin and ethylene-mediated responses [13].

Mulberry is grown in a diverse range of climates including temperate, subtropical to tropical conditions. Its leaves are the sole food source for the silkworm on which the silk industry thrives. The dietary preference of silkworms has been attributed to the traits of the leaf including the presence of the optimal amount of certain sterols including beta-sitosterol and ergosterols [14]. Another study reports that the leaves with lower crude fibre, fewer ideoblasts and cystoliths, and fewer mucilaginous cells are preferred by the worm [15]. By virtue of its habitat and genotype, various accessions of mulberry regularly face abiotic and biotic stresses. These stresses take their toll on the ultimate biomass yield which severely impacts the silk industry. As per estimates, there are about 200 insect and non-insect pest species that are known to attack mulberry. The pink mealy bug (*Maconellicoccus hirsutus* Green), papaya mealy bug (*Paracoccus marginatus*), leaf webber (*Diaphania pulverulentalis* Hampson) and thrips (*Pseudodendrothrips mori* Niwa) are some of the major pests which cause a loss in mulberry leaf yield. Mulberry is also very prone to water deficit stress [16]. Salinity stress is also known to hamper the yield of mulberry leaves. Efforts are being made to raise a vigorous hybrid by crossing different cultivars. Efforts are also being made to harness the genetic diversity in the different species of *Morus* to find genes which could have positive implications for stress mitigation. *Morus laevigata* (ML) and *Morus serrata* (MS) are two wild relatives growing widely in India. ML shows a wider range of adaptability and is distributed throughout the country. Although it has been traditionally utilized as a source of timber and forage [17], it is also recognized as a good source of stress mitigating genes [18]. On the other hand, MS which is also known as Himalayan Mulberry, is restricted to the relatively homogenous environmental condition of the Himalayan foothills. MS along with ML are known for their resistance to certain pests including *Phyllactinia corylea* [19]. *Morus notabilis* (MN) is native to the Sichuan province of China and is widely acclaimed for having a battery of genes conferring resistance to biotic and abiotic stresses [20]. Genome sequencing of an organism provides the opportunity to analyze the number of genes in a family and their evolutionary pattern in a more robust manner. RNA sequencing from different developmental stages combined with the genome sequence gives a direct indication of expression dynamics. Furthermore, a particular gene differential expression in contrasting genotypes indicates the potential contribution to their thriving. The expression of all the identified ARFs was analyzed in three different genotypes by qPCR experiments in drought and indole-3-acetic acid (IAA) treatments. They showed varied responses to these treatments in different genotypes. Such differential regulation of regulator genes in plant systems has adaptive value and these genes could be targeted for the improvement of different cultivars. We therefore utilized the published draft genome to study ARF gene family members in functional and evolutionary contexts. With a wide range of roles, such as plant growth and development and abiotic stresses, ARFs could be important transcription factor components which could be studied and targeted for the varietal improvement of mulberry via a transgenic approach. In this study, we have made a detailed analysis of ARFs, including a structural and functional analysis. We have studied their expression profile and compared them in leaf tissues of three wild relatives including *M. laevigata*, *M. serrata* and *M. notabilis*. In this study, we have made a collective effort to catalogue the ARF coding genes in mulberry, investigate their expression profile and study phylogenetic relationships which could eventually be a useful resource for the mulberry research community.

## 2. Materials and Methods

### 2.1. Plant Material

Mature leaves of *M. serrata* (Department of Plant Molecular Biology, South Campus, University of Delhi, New Delhi, India) and *M. laevigata* (Department of Botany, North Campus, University of Delhi, New Delhi, India) were harvested. These were flash frozen in liquid nitrogen and stored at −80 °C in a deep freezer until further use.

### 2.2. Transcriptome Assembly, Functional Annotation and Differential Expression Analyses

RNA-Seq reads for *M. notabilis* for five samples were downloaded from MorusDB [21]. The NCBI SRA accession for the samples is SRX504963, SRX504944, SRX504924, SRX504906 and SRX504893 for leaf, male flower, winter bud, bark and root tissue respectively. Our group has previously sequenced and submitted the raw data [22,23,24] for ML and MS with accession numbers SRX1515878 and SRX1506562 respectively. Trinity software version r20140413p1 [25] with default parameters was used to assemble the sequences of *M. serrata*, *M. laevigata* and *M. notabilis*. A preliminary quality assessment of these reads was done using Fastqc version 0.11.2 [26]. Trimmomatic version 0.30 [27], which is provided as a plugin, was used to remove the adapter sequences, primer sequences and their fragments along with those reads which were not up to the mark, with the default settings LEADING:5, TRAILING:5 and MINLEN:36. The sequences were then assembled using Trinity with in silico read normalization. For functional annotation of the assembled transcripts, the Fastannotator (Chan Gung University, Taoyuan, Taiwan; [28]) server was used. Briefly, it uses BLASTx search against nr database and BLAST2GO suite for assigning gene ontology terms. Assembly generated using Trinity [25], using all seven RNA-seq files, was used as a reference. The Align_and_estimate_abundance.pl script provided in the Trinity software which utilizes bowtie2 [29] was used to estimate the abundance in the seven developmental stages. Differential expression analysis was carried out in R using the edgeR package [30] by utilizing the run_DE_analysis.pl and analyze_diff_expr.pl Perl script provided in the same suite. To identify ARF members from the assembly, the tBLASTn program from the NCBI blast suite 2.2.28+ was used and the resultant best hits satisfying significant *E* value (*E* value ≤ 10^−10^) and sequence similarity scores were checked for the presence of conserved domains.

### 2.3. Identification and Phylogenetic Analyses of ARFs

The proteome of *M. notabilis* was downloaded from MorusDB [21]. The ARFs amino acid sequences were also downloaded for *Arabidopsis* (https://www.arabidopsis.org) and banana (http://banana-genome.cirad.fr/) [31]. A Hidden Markov Model (HMMER: http://hmmer.janelia.org/) built from known ARFs with accession number PF06507.8 was fetched from PfamA.hmm, supplied by the Pfam database (http://pfam.xfam.org/) using the HMMfetch program provided with the HMMER suite version 3.12b. This hmm profile was used to search the proteome of *M. notabilis*. The resultant sequences were searched for ARF domains using NCBI CDD (http://www.ncbi.nlm.nih.gov/Structure/cdd/cdd.shtml) [32]. The identified ARFs were included in the new hmm profile and the search was repeated until no new members could be identified. A parallel BLASTp search was also made using *Arabidopsis* and banana ARFs as a query against the *M. notabilis* proteome. The resultants were checked for conserved ARF domains using NCBI CDD. For the phylogenetic studies, MEGA version 6.06 was used [33]. A maximum likelihood phylogeny reconstruction analysis was conducted and the inferred phylogeny was tested with 1000 bootstrap replications. The resultant phylogenetic tree was edited using Figtree Ver 1.4.2 (http://tree.bio.ed.ac.uk/software/figtree/).

### 2.4. Structural and Functional Analyses

The corresponding mRNA and genomic region sequences encoding for these identified ARF proteins were fetched from the scaffolds of Morus present at MorusDB. The gene structure display server (http://gsds.cbi.pku.edu.cn/) was used to show the structural arrangement of exons and introns. This was also confirmed by the coordinates given in the sequence annotation on the MorusDB server [21]. Molecular mass estimation and isoelectric point estimation was done using a Perl written script employing bioperl modules [34]. Motifs present in the sequences of identified ARFs were searched and analyzed using the MEME suite (http://meme-suite.org/). The number of motifs searched was 15 and the optimum motif width kept was between 8 and 50 residues. Furthermore, these predicted motifs were annotated by using the InterProScan database search (http://www.ebi.ac.uk/Tools/pfa/iprscan/). Gene ontology terms for the sequences were fetched and an enrichment analysis (hypergeometric test) with Benjamini and Hochberg false discovery rate correction was performed using the BINGO plugin [35] and a network displaying enriched terms was generated in Cytoscape version 3.02 [36]. The String-db protein network [37] analysis tool was used to get the interaction network based on the model plant *Arabidopsis*.

### 2.5. Promoter Analysis, cis-Elements Identification and Their Enrichment Analysis

2 kb upstream regulatory regions (URRs) for all the predicted genes in MorusDB [21] were extracted using a custom written Perl script employing samtools. URRs of ARFs were taken and searched for *cis* elements using the *cis* element database from PLACEDb [38]. For identification of significantly enriched transcription factors, the binding site Pscan [39] was used locally against the JASPAR database [40]. A significance cut-off of *p* value ≤ 0.05 was applied to report the significantly enriched TF binding sites in ARF URRs. Nine important *cis* elements pertaining to hormonal and stress regulations were shown using a diagram generated using a custom written GFF file.

### 2.6. Stress and Hormonal Treatments and Relative Expression Analyses with qPCR

To understand the differential regulation in response to stress and hormonal treatments, quantitative PCR (qPCR) based expression profiling was done for all 17 identified ARFs. For the qPCR experiments, fresh leaf tissues from field grown mature mulberry plants of three genotypes including ML, MS and MI (*Morus indica*) were harvested. Four conditions were designed including Control 0 h, Control 4 h, desiccation treatment and IAA treatment. The experimental setup described previously [41] was followed. Control 0 h represents the samples that were snap chilled and stored in liquid nitrogen immediately. Control 4 h represents the samples kept in reverse osmosis water for four hours at 28 °C. Drought treatment was given by transferring the same setup to 0.5 M mannitol solution and keeping it at 28 °C for four hours. 100 µM solution of 3-Indoleacetic acid (Sigma-Aldrich, Steinheim am Albuch, Germany) was mixed in the same setup and kept for 4 h at 28 °C. After these treatments, treated tissues were snap chilled in liquid nitrogen and stored at −80 °C for further usage. The total RNA samples from two biological replicates were isolated using a modified GITC protocol [42]. After isolation, samples were treated with DNase and column-purified using the RNeasy Plant Mini Kit (Qiagen, Hilden, Germany). These samples were checked for qualitative and quantitative parameters using a spectrophotometer (NanoVue; GE Healthcare, Buckinghamshire, UK) and denaturing agarose gel. To synthesize cDNA from these samples, a high capacity cDNA archive kit (Applied Biosystems, Foster City, CA, USA) was used. 2.0 µg of the total RNA was taken from each sample in a 50 µL reaction and instructions from the manufacturer were followed strictly. To amplify the ARF genes, primers were designed in Primer Express software version 2.0 (Applied Biosystems) with default parameters. A list of the primers is given in supplementary Table S1. The uniqueness of the primers was checked by BLAST and searched against the combined assembly and melt curve analysis after the run was complete. The PCR reaction was performed in the ABI Prism 7000 sequence detection system (Applied Biosystems). Two biological replicates with three technical replicates for each sample were taken and Actin was used as an internal control. The estimation of relative expression values was undertaken using the 2^−ΔΔCt^ method. Student’s *T* test was employed on ΔCt values of different biological replicates of control 0 h and treatments to test the statistical significance (*p* value ≤ 0.05).

### 2.7. Interaction Networks of Mulberry ARF Genes

Mulberry ARF sequences were used as a query sequence in the String-db database [37] with *Arabidopsis* as the model. The nearest homologs of the mulberry ARFs were taken and their interacting partners in *Arabidopsis* were identified, which could have interaction evidence. These interacting partners were searched in the combined assembly with the BLASTp tool and their expression profile was assessed in the same manner as for the ARF genes.

## 3. Results

### 3.1. Structural Features of Mulberry ARFs Encoding Genes and Proteins

**Figure 1:**
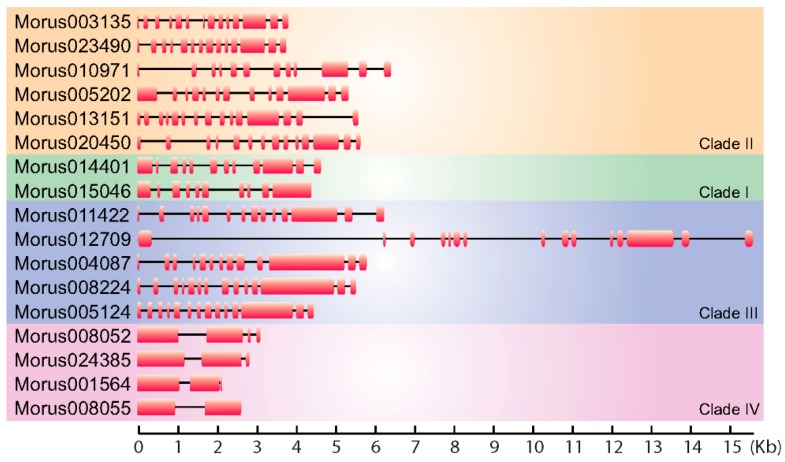
Gene structure diagram showing the arrangement of exons and introns of the *Morus notabilis* Auxin Response Factors (ARFs). Exons are shown using solid boxes and introns are represented using lines. Genes are separated into four groups as per their phylogenetic distribution.

A total of 17 ARFs encoding genes have been identified in the *M. notabilis* genome. BLAST similarity and HMM search methods were employed to search the whole proteome of mulberry. The ARF nature of these proteins has been confirmed by the presence of conserved ARF motifs in an analysis employing NCBI CDD. The size of genes encoding these ARFs in mulberry ranged from 15,593 bp to 2152 bp (Figure 1). Their coding sequences (CDSs) size ranged 1839 bp to 3435 bp and the number of exons and intron ranged from 2 to 15 exons. *Morus008055* was found to encode a protein of 622 amino acids length, identified with a single intron. These ARFs ranged from 612 to 1144 amino acids in length (Table S2), however in banana ARFs range from 448 to 1067 [43]. The smallest ARF encoding gene identified in *Morus* is *Morus001564*. The molecular masses of these ARFs proteins ranged from 52.59 kDa to 101.10 kDa. The isoelectric point (pI) for these proteins ranges from 4.86 to 8.41 suggesting a broader range of activity in microcellular environments. Furthermore, only two of them (Morus005202 and Morus008052) showed a basic pI while the rest showed an acidic pI. To identify the conserved motif in these proteins the meme-motif search tool was used [44]. Among the predicted conserved motifs identified in these ARFs were the DNA-binding pseudo barrel domain, the ARF domain and PB1 domains. These are the signature domains of ARF proteins and have important biological functions. Several other domains of unknown function were also identified as fairly conserved. However, functions performed by them are not known.

### 3.2. Phylogeny and Classification of Mulberry Auxin Response Factors

**Figure 2:**
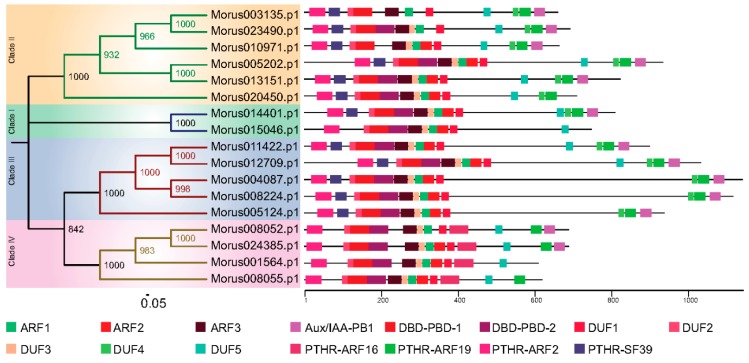
The phylogenetic tree of *Morus* ARFs. Four clades are highlighted with different coloured backgrounds. The conserved domains of the protein are shown using different coloured boxes (See legend for details). The numbers on the nodes of the tree depict the bootstrap values obtained.

The phylogeny based classification has been proposed for many gene families. It has been utilized to classify ARF genes in various systems including banana [43] and *Arabidopsis* [6]. To study the evolutionary pattern among *Morus* ARFs, a maximum likelihood phylogenetic tree was constructed using the amino acids sequences of 17 members. In this analysis, *Morus* ARFs separated into four clades with a very high bootstrap confidence values (Figure 2). In the members of clade I, there are 10 to 12 exons and 9 to 11 introns present. In clade II, members have 12 to 15 exons and 11 to 14 introns. Clade III, which consists of five members, has 13 to 15 exons and 12 to 14 introns. Clade IV members have a comparatively lower number of exons and introns. Exons in this clade range from two to four, with one to three introns. Two variants of DNA-binding pseudo-barrel domains were predicted as conserved domains in meme motif analysis and annotation by Interpro. Type 1 DNA-binding pseudo-barrel (DBD-PBD-1) was present in all 17 ARFs while the second type was present in all but Morus003135 and Morus010971. The PB1 (Phox and Bem1) domain is considered an integral part of ARF and AUX/IAA proteins and is usually present in the C-terminal of the protein. These domains are involved in protein interaction and required for heterodimerization or homo-oligomerization. Except for four ARFs (Morus015046, Morus020450, Morus001564 and Morus008055), all contain this domain. These genes belong to three of the four clades, all except clade III. AUX/IAA domains are present in three of the four clusters namely I, II and III. It was shown earlier that those ARFs that lack these PB1 domains are termed as truncated ARFs. For these truncated proteins, detailed functional investigations are lacking except for ETTIN and ARF4 which have been shown to be involved in carpel development [5]. Thus, our analysis found that the mulberry genome also encodes for truncated ARFs and these truncated ARFs could be used to study the functional roles played by these truncated proteins. Interpro also reports the classification system proposed by PANTHER [45]. Interpro reported the absence of the PTHR31384:SF39 domain from all the members of group IV. This particular motif was also absent from Morus015046, a group I member. Group IV was also unique as it exclusively contains the PTHR31384-ARF16 domain. In meme motif searches five highly conserved motifs were identified. These were named as DUFs because they could not be annotated. DUF 4 was found to be absent from group IV. It may have been of certain evolutionary significance and could be potentially used as a separate group IV member, distinct from those in other organisms.

**Figure 3:**
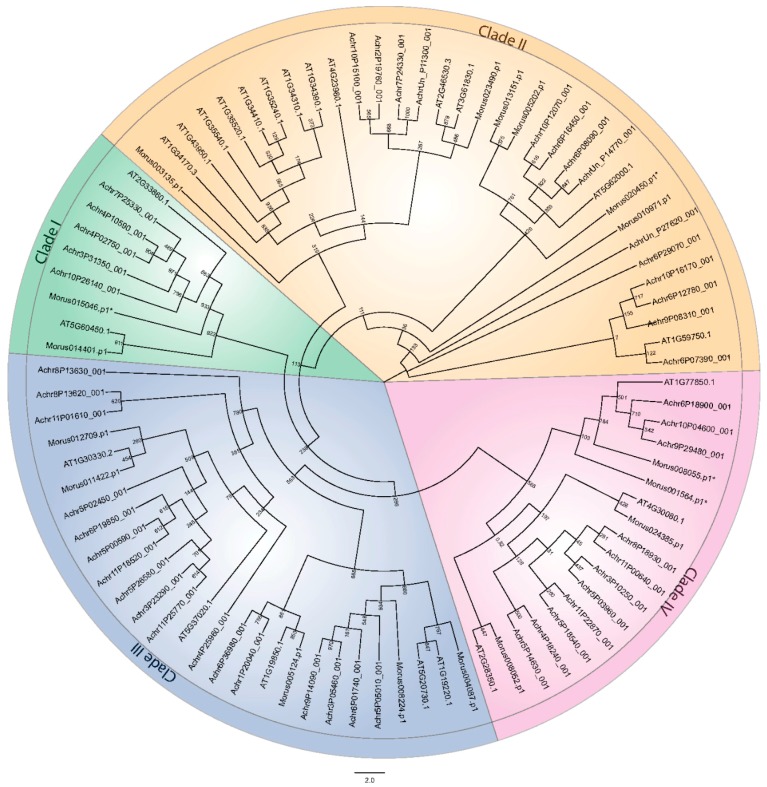
Cladogram depicting the phylogenetic distribution of the ARFs of *Morus*, *Arabidopsis* and banana into four clades. Phylogenetic analysis was conducted with 1000 bootstrap replicates. Clades are shown by different colored backgrounds. * represents the truncated ARFs.

To assess the evolutionary origin of these members, phylogenetic analysis was conducted along with the *Arabidopsis* and banana ARFs. These samples were taken as being representative of dicot and monocot systems respectively. Sequences from all three genera were aligned. The alignment was used to generate a cladogram depicting the evolutionary history of these ARFs. As it was observed in other systems, ARFs in mulberry were also segregated into four separate clades (Figure 3). The presence of *Morus* and *Arabidopsis* ARFs along with banana ARFs in the same monophyletic clades suggests co-evolution before the separation of monocot and dicots lineages. Clade I consists of only two members, Morus014401 and Morus015046. This clade is also identified as having evolved recently as suggested by the relatively long branches of the tree. Clade II contains six members and most of them separated with *Arabidopsis* members. Similarly, five ARFs are found in clade III and in clade IV, four members are well separated. Two Truncated ARFs of mulberry belonging to clade IV formed a clade along with three banana ARFs and AT1G77850.1 (ARF17) of *Arabidopsis* which is also a truncated ARF [46]. Similarly, another truncated ARF of mulberry was found to form a clade with AT2G33860.1 (ETTIN/ARF3), again a truncated ARF [46,47]. It was argued that ETTIN and ARF4 were the product of a duplication event from a non-truncated ARF gene [5] and the lineage continued as such which is also supported by our findings.

### 3.3. Distribution of cis Elements in 2 kb Upstream Region and Gene Ontology Enrichments Analysis

**Figure 4:**
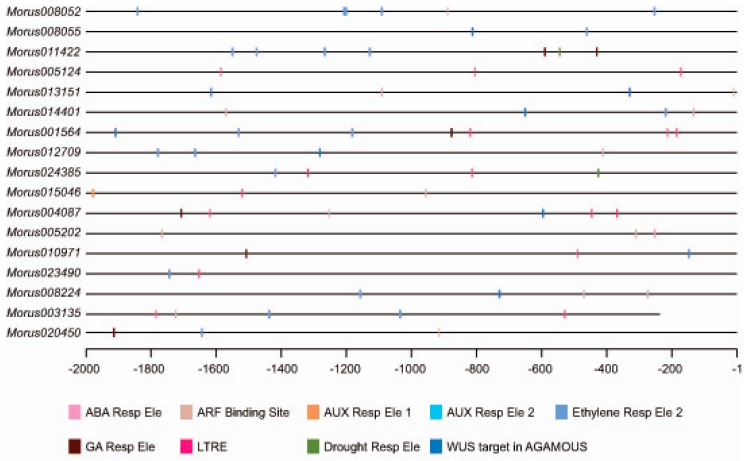
Distribution of *cis*-elements in the putative promoter region of *Morus* ARFs genes. The putative promoter region is shown using a straight line. Different types of *cis* elements are shown using different colors which are given as a legend.

Most of the transcription factors are known to bind to specific *cis* elements and bring modulation in the expression of underlying genes. Analysis of *cis* elements in the upstream regulatory region (URR) of a gene can give a clue about the regulatory regime it follows. We analysed the 2 kb upstream of all identified ARFs in mulberry. The *Morus003135* promoter falls in the sequencing gap region and contains artificially filled ambiguous *N* nucleotides. This was removed from the analysis thus effectively bringing the size of its promoter to 1764 bases. Analyses of *cis* motifs in putative promoters revealed that these ARFs possess important *cis* regulatory motifs such as ARF binding sites, ethylene responsive elements and drought responsive elements (ACCGAGA) etc. In total, 71 such *cis* elements were identified in promoters of these ARFs in mulberry (Figure 4). The three most highly represented *cis* elements were the ethylene responsive element (21), the low-temperature response element (16) and the ARF Binding site (17). The Auxin Response Factor binding site (TGTCTC) was found in 10 out of the 17 members. Morus013151, Morus014401, Morus005202 and Morus008224 have two sites each while all of the others have a single such binding site in their 2 kb URR. The ethylene responsive element (AWTTCAAA) [48] was also found in 10 ARFs and five of them contain more than one such element. Auxin responsive elements were found in 2 kb URR of Morus015046. Gibberellic acid responsive elements were found in the URR of five ARFs namely *Morus011422*, *Morus001564*, *Morus004087*, *Morus010971* and *Morus020450*. It is possible that these ARFs might be involved in the hormonal crosstalk because of the presence of such hormone responsive motifs. The URRs of two genes i.e., *Morus011422* and *Morus024385*, possess the Drought Responsive Element 1 (DRE1) element (ACCGAGA) which was earlier found in the promoter of the *Rab17* gene and was responsive to increased abscisic acid (ABA) and drought treatments [49]. Interestingly, the URRs of eight ARFs possess a *cis* binding site for the *WUS* gene which was earlier reported in the *AGAMOUS* intron [50]. This interaction is shown to regulate the floral transition. Possession of these elements indicates a possible role of these ARFs in mulberry for the same process.

Pscan [39] was used to find over-represented transcription factor binding sites in the URRs of ARFs. The transcription factors binding site available from JASPAR database [40] has been used for this analysis which contains matrices of 1082 binding sites. In this analysis, 78 such binding sites were found to show significant (*p* ≤ 0.05) presence when tested against the whole genome promoter regions (Supplementary Table S3). This list includes binding sites for Zn fingers, Squamosa and AP2 proteins etc. Most of these transcription factors have shown an important role in growth and development, cell and tissue patterning, and controlling new organ formation etc. It is possible that ARFs under the influence of these transcription factors might regulate these aspects in mulberry.

**Figure 5:**
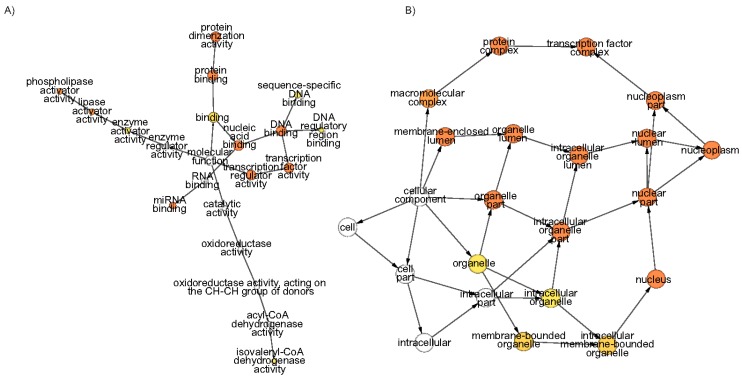
The network of the significantly enriched gene ontology (GO) terms of *Morus* ARFs. (**A**) Molecular Function and (**B**) Cellular Components. The color of the nodes shows the significance level. The hypergeometric distribution test with Bonferroni correction was applied. Node size and colour intensity represent abundance and significance level, respectively.

Gene ontology (GO) terms are like a niche for a gene which gives an idea about its function, mode and whereabouts. GO terms cover three domains and for ARFs, representations are from all three. In enrichment analysis, significantly enriched GO term in the biological process contains the auxin mediated signalling pathway GO:0009734. Another significant GO term GO:0048438; floral whorl development was identified along with several GO terms related to floral organ development. The GO term GO:0006355; DNA-dependent regulation of transcription was also significantly enriched in accordance with the structural moieties of these proteins. Production of Trans-acting siRNA (ta-siRNAs) involved in RNA interference (GO:0010267) was also identified supporting the recently validated fact that tasiRNA-ARF has a role in floral morphogenesis [51]. Besides other important biological processes, GO terms including root morphogenesis, embryonic axis specification, etc. are also highlighted (Supplementary Figure S1 and Table S4). In molecular function GO terms, protein dimerisation activity, DNA binding activity, transcription factor activity and miRNA binding activity were significantly enriched suggesting the diverse molecular role played by these ARFs (Figure 5A and Table S5). In cellular components GO terms, signifcantly enriched ones are protein complexes, transcription factor complexes, the nucleoplasm and nucleus are the GO terms which are significantly enriched (Figure 5B and Table S6).

### 3.4. Expression Profiles of Auxin Response Factors in Developmental Stages of Morus Notabilis

**Figure 6:**
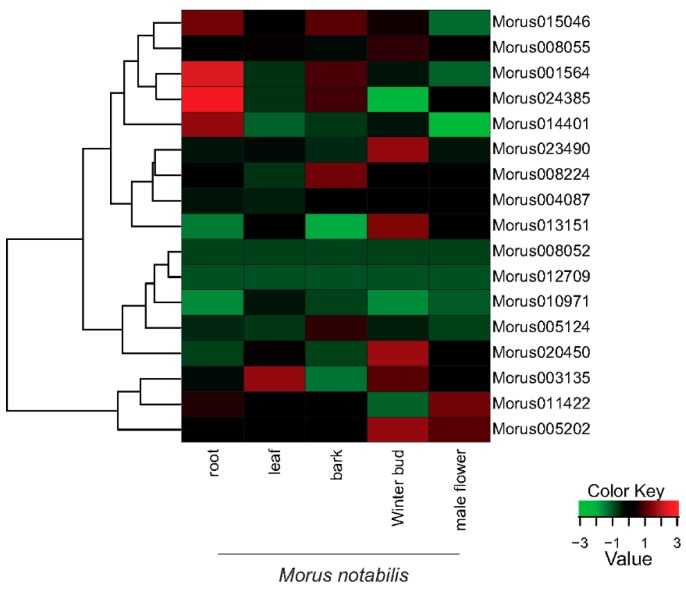
A heat map showing the relative expression profiles of the identified 17 ARFs across the five developmental stages of *Morus notabilis*. FPKM values (median centre normalized) for all stages were used. Green represents down regulation while red represents up regulation (See legend for details).

The expression profiles of the 17 identified ARFs showed a vivid range of expression spanning five tissues of *M. notabilis. Morus0101564* and *Morus024385* have shown the highest expression in root tissue followed by *Morus015046* and *Morus014401* (Figure 6). The trimmed mean of the *M* value normalization method was applied [52]. FPKM values (Fragments Per Kilo-base of transcript per Million mapped reads) were used to assess the expression levels across the stages. *Arabidopsis ARF19* and *ARF7* have been shown to express in a root specific manner [13]. ARFs in bark tissues are moderately expressed. Only four ARFs have shown expression towards higher ranges in bark tissue including *Morus015046*, *Morus024385*, *Morus0101564* and *Morus008224*. There are various studies which have established the role of ARFs in fruit development. ARF9 of *Solanum lycopersicum* has been shown to express in early fruits and regulate the cell division activity [53]. Winter bud, which represents the early fruit stage, is the tissue where a maximum number of ARFs (seven) have shown to express in higher ranges. These include *Morus005202*, *Morus003135*, *Morus020450*, *Morus013151*, *Morus023490*, *Morus008055* and *Morus015046*. These genes might be playing a significant role in the fruit development of *Morus*. The only highly expressed ARF of leaf tissue is *Morus003135*. It has the highest expression value in leaf tissue among the stages studied. *ARF7* in *Arabidopsis* has an established role in regulating auxin response genes in leaf tissue [54]. The expression profile of *Morus003135* could be an indicator of its role in leaf development. In the male flower, two ARFs including *Morus011422* and *Morus005202* are highly expressed. *ARF6* and *ARF8* have a well-documented role in floral development. The mutation of these genes in *Arabidopsis* caused delayed stamen development and decreased fecundity [55]. Hence it might be speculated that *Morus011422* and *Morus005202* could be playing an important role in male flower development in *Morus*. The expression of the two ARFs *Morus008052* and *Morus012709* is very low in all the stages included.

### 3.5. Differential Expression of ARF Genes in Three Wild Species of Morus

**Figure 7:**
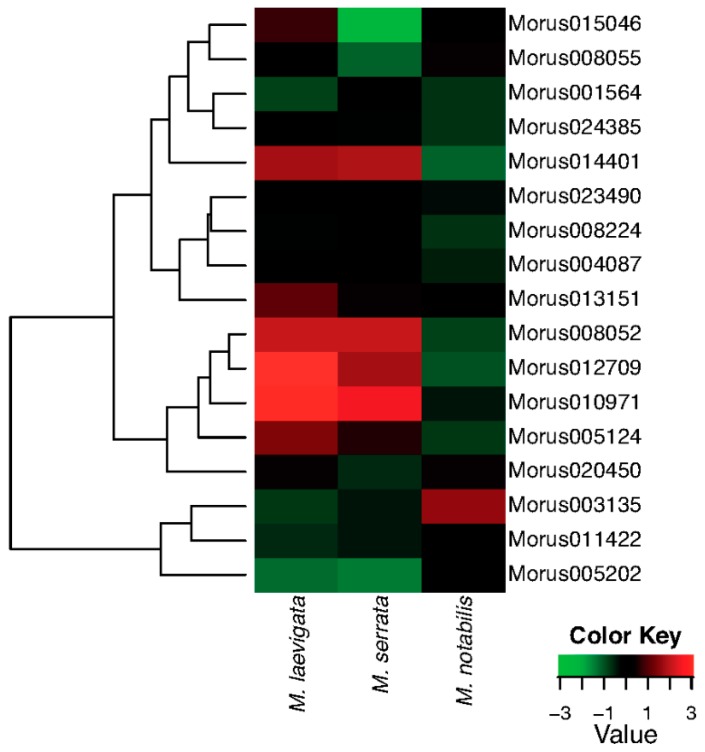
A heat map showing the hierarchically clustered comparative expression levels of Auxin Response Factors of *Morus* in three different species i.e., *Morus notabilis*, *Morus laevigata* and *Morus serrata*. FPKM values (median centre normalised) were used to plot this heat map.

The leaf forms the most important tissue of the mulberry plant as it is the ultimate economic source which is used as feed for the silkworm. *M. laevigata* and *M. serrata* are the species which have very important traits pertaining to leaf quality. ARFs expression profiles have been compared across the three leaf tissues of three different *Morus* species (Figure 7). Out of 17 ARFs, four have very high and similar expression patterns in *M. laevigata* and *M. serrata* leaves, while in *M. notabilis,* these genes are expressed at relatively low levels. Furthermore, two ARF genes—*Morus013151* and *Morus005124*—are expressed with higher expression values in *M. laevigata* only. *Morus003135* has shown higher expression in the *M. notabilis* leaf compared to the other two species. *Morus008052*, *Morus005124* and *Morus012709* are three genes identified as having light responsive elements in their 2 kb upstream regulatory region. These three genes are expressed differentially in these three tissues with the highest expression value in *M. laevigata* followed by *M. serrata*. Distribution of these three species is varied as *M. laevigata* is found to grow in all terrains from the Terai belt of the Himalayas to Andaman Nicobar in India while *M. serrata* is restricted to the highland of the Himalayas [18,56] and *M. notabilis* is grown in the Sichuan and Yunnan provinces of the People’s Republic of China where the climate is a subtropical highland type. It might be possible that these ARFs genes could be playing some role in their local adaptation via physiological and morphological responses. A Supplementary Table (Table S7) is given with FPKM values for all the seven stages used in this study.

### 3.6. Genotype Specific Differential Expression of ARFs in Drought and Hormonal Treatment

**Figure 8:**
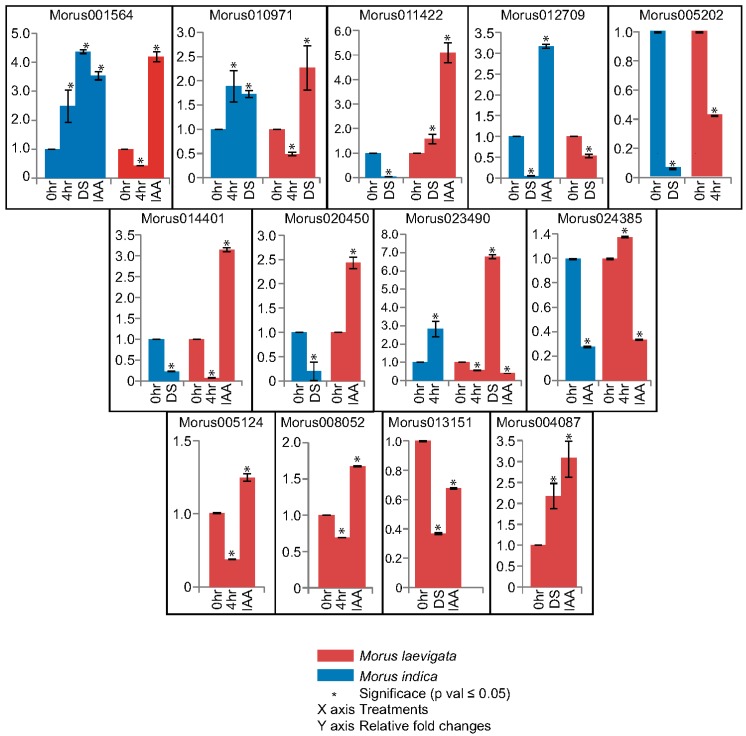
Real time PCR based relative fold changes assessment of ARFs in response to stress and hormonal treatments. Different genotypes are shown using different coloured bars. Error bars are plotted using the standard error of two biological replicates. Three technical replicates were run for each biological replicate. The asterisk shows that the fold changes calculated are significant (Students’ *T* test; *p* value ≤ 0.05). DS: desiccation; IAA: indole-3-acetic acid.

In qPCR experiments, 13 out of 17 ARFs have shown differential expression under drought stress and hormonal treatment (Figure 8). This study was done in three contrasting varieties including ML, MS and MI which are known to flourish in different geographical regions and climates. *Morus indica* is a cultivated variety known for its profuse flowering and fruiting. It is also known for its robustness and widely prescribed for cultivation in a wide variety of conditions. *Morus001564* and *Morus010971* are the two genes that have shown significant up-regulation in drought stress treatment in MI. Although in URR analysis, these genes did not show the presence of drought responsive elements. *Morus001564* and *Morus012709* genes have shown up-regulation in IAA treatment in the same genotype. *Morus012709* possesses an ARF binding site and no auxin response element in its URR. This suggests that it may be regulated indirectly by some other ARF. *Morus012709*, *Morus005202*, *Morus014401* and *Morus020450* are ARFs that have shown down regulation in response to drought stress treatment in MI. *Morus024385* was the only gene that has shown down regulation in response to drought stress in the same genotype. However, in the URR of this gene a drought responsive element was reported. It might be possible that in MI, this drought responsive element is not functional. Compared to this, the relative expression profile of these genes varies in ML. In ML leaf tissue, *Morus001564* was up-regulated in IAA treatment only. Compared to the MI expression pattern, *Morus011422* in ML was up-regulated in both drought stress and IAA treatment. *Morus011422* contains a drought responsive element and this is possibly functional in ML. Similar to MI, *Morus012709* was significantly down regulated in ML as well as in response to drought treatment. Two genes, namely *Morus014401* and *Morus020450*, have shown genotype specific (ML) up-regulation in response to IAA treatment. These two genes were found to possess ARF binding sites in their URRs. Their up-regulation by IAA treatment indicates the possible functional nature of these elements. Similarly, two genes i.e., *Morus023490* and *Morus024385* have shown ML specific down-regulation in response to IAA treatment. Contrary to this, *Morus023490* has shown a strong up-regulation (>6 fold change) in response to drought stress in ML. A noteworthy observation in this analysis was made regarding genotype specific regulation of ARFs in ML only. Four such ARF genes—*Morus005124*, *Morus008052*, *Morus013151* and *Morus004087*—have shown exclusive differential expression in ML only. All but *Morus013151* have shown up-regulation in response to IAA treatment. The varying responses to different treatments could thus be attributed to no functionality or acquisition of such motifs during evolutionary course. Previous studies have also reported a vast number of SNPs and InDELs in these three genotypes [23].

### 3.7. Interaction Network of Mulberry ARFs

**Figure 9:**
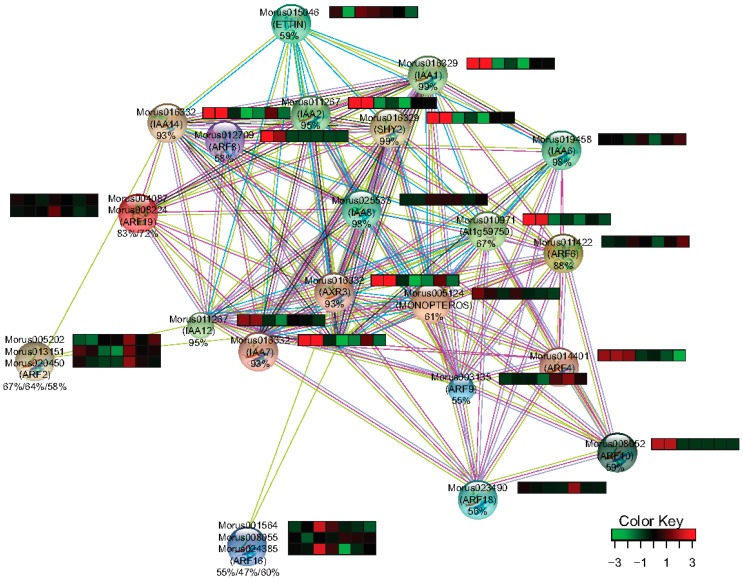
The interaction network and expression profile of the identified interacting partners of ARFs in *Morus*. Heat maps show the expression level (FPKM values) across the seven stages used in this study. The percentage identities of the *Morus* genes with *Arabidopsis* genes are shown below the IDs. From left to right the stages are *Morus laevigata* leaf, *Morus serrata* leaf, *Morus notabilis* root, bark, winter bud, leaf and male flower.

The interaction network is one of the key utilities for gaining insight into the function of a gene family. Homology based interpolation of the interaction network has been utilized to substantiate the interaction in other organisms as well [43]. String-db [37] was utilized to get the information about the interacting partners of *Morus* ARFs. 12 ARFs of *Arabidopsis* were found to represent all 17 ARFs of mulberry with different levels of similarity ranging from 47–88%. In the interacting network thus created, 12 ARFs, 9 IAA including SHY2 which encodes for IAA3, and AXR3 encoding for IAA17 were the protein nodes that were identified (Figure 9). *Arabidopsis* IAAs homologs were searched in *Morus* using the BLASTp approach and their differential expression profile in the studied stages was extracted from combined assembly. Nine IAAs identified in this network were represented by only five unique transcripts in de novo assembly and all five of them were differentially regulated in the studied stages. IAA14 and IAA12 which have been shown in the network to interact with ARF2 (Morus005202.), have a similar profile and somewhat of a correlation with ARF2. The stages with higher ARF2 expression showed the accumulation of transcripts of its interacting partners are lesser. Similarly, ETTIN (Morus015046) which is shown to interact with IAA1, IAA2 and IAA14 has a contrasting expression profile suggestive of a possible regulation at transcript level. MONOPTEROS is known to be regulated by a feedback loop and also regulates the expression of its interacting partner IAA12 in *Arabidopsis* [57]. Thus, expression patterns observed for ARF2 and ETTIN and other ARFs also warrant a similar kind of possible regulation in mulberry.

## 4. Discussion

Mulberry is one of the most important trees, particularly for the silk industry. The monophagous silk insect feeds on it and a direct correlation between the quality of leaves of the mulberry tree and silk output has been observed. ARFs are important regulators of various plant growth and stress responses. They act by regulating the expression of downstream transcription factors and other genes. The ARFs bind to specific *cis* elements (AuxRE, ARF binding sites) and are known to regulate downstream genes. Mulberry species are susceptible to various types of biotic and abiotic stresses which take a toll on leaf growth and development. Furthermore, some wild species of mulberry show robust and better leaf development and their developmental stages show higher regulation of genes contributing to resistance [58]. ARFs have been known to regulate such aspects thus could have a direct implication in mulberry improvement programs.

Genome sequence availability provides an opportunity to study this important gene family in mulberry and the availability of transcriptome sequences from different stages of development from the same genotype and leaf stages provides an important resource to compare the expression profile of these genes in contrasting varieties. In this study, ARF encoding members of mulberry have been identified. Mulberry contains a total of 17 such genes which is the second least (*Carica papaya* with 11 ARFs is the least [59]) in systems studied so far and similar to *Eucalyptus grandis* [8]. Similar to other systems, mulberry ARFs are also classified in four clades with specific structural aspects. Similar to other species, the fourth clade of mulberry ARF also possesses the least number of introns. These ARFs fulfil all the requirements of having an N-terminal DNA-binding domain, a C-terminal dimerisation domain and a variable middle region. Like *Arabidopsis* and other monocots, mulberry also have truncated ARFs. Truncated ARFs in mulberry are four in number and in the phylogenetic study, these truncated ARFs formed a clade with *Arabidopsis* truncated ARFs. This observation further strengthens the hypothesis of their origin through duplication of truncated ARFs only. A very high level of conservation among the members is also revealed in phylogenetic analysis by bootstrap values. This suggests the fairly conserved nature of these genes across dicot model system *Arabidopsis*. In phylogenetic analysis, the majority of these ARFs in mulberry formed a clade with *Arabidopsis* ARFs, suggesting the separate origin of these ARFs. Putative promoter sequences of these genes contain important *cis* elements like auxin responsive elements and motifs required for light mediated responses etc. Some of the ARFs responded to drought stress and IAA treatment in qPCR experiments and sometimes they deviated from the predicted *cis* elements in their 2 kb URRs. The possibility of these elements absence or acquisition during the course of evolution could be a possible answer for the differential responses of different genotypes. An array of important transcription factor (TF) binding sites including AGL, Zn fingers etc. has shown significantly biased presence in ARFs URRs. This suggests that a wider possible role might be played by ARFs in the growth and development of mulberry. GO enrichment analysis of these ARFs revealed wider functional aspects of these genes and supports their role which has been established in other systems. This includes transcription factor activity, a role in floral development, stress and root morphology development etc. Furthermore, expression analysis in five developmental stages also revealed its diverse range of expression. *Morus001564* and *Morus024385* showed higher expression in root tissue and they might be shaping and regulating the root morphology. ARF9, 14, 16 and 17 in *S. lycopersicum* have been shown to be expressed at a relatively high level in root tissue [60]. Five ARFs including *Morus023490*, *Morus013151*, *Morus020450*, *Morus003135* and *Morus005202* have a very high expression in winter buds and might be involved in floral development. Dormancy breaking also requires active modulation of auxins [61] and ARFs might play a role. Furthermore, in comparative transcriptome analyses, several ARFs in three wild relatives showed different expression. This could be due to the possibility of acquired regulatory elements and modules pertaining to a particular climate. Some of the ARFs have shown higher expression in ML and MS leaves compared to MN leaves. These three species vary broadly in anatomical, morphological and agroclimatic attributes. The differential expression profile exhibited by ARFs in these three genotypes in the leaf tissues might be one of the regulatory components shaping these climate-related genes. Furthermore, differential expression was reported in three genotypes including ML and MI. So, the same gene in different genotypic background behaved differentially in response to various treatments. This indicates the possibility that these genes either are regulated by different trans factors, or that some of the *cis* element modulations have taken place. Thus, taken together our analysis provides a strong foundation for further studies of these ARFs in the mulberry system.

## 5. Conclusions

In this analysis, 17 ARF coding genes of mulberry have been identified. These 17 genes were classified in four clades based on their protein sequence based phylogenetic analysis. Furthermore, all the ARFs have shown the hallmark domains thus confirming their ARF nature. Four truncated ARFs were also identified in mulberry and they have made clades with truncated ARFs of *Arabidopsis*. GO enrichment analysis coupled with the presence of known *cis* elements in their 2K upstream regions revealed a wider array of domains where these genes might work. This includes floral development, stress regulation, organogenesis, morphological regulation and leaf, root and shoot development etc. In expression analysis these ARFs were found to be regulated in a tissues-specific manner. RNA-Seq based analysis revealed tissue specific expression of these ARFs. Root and floral bud preference expression has been observed suggesting their possible roles in establishment of these tissues. In the qPCR based analysis, these ARFs were shown to be differentially regulated in response to auxin and drought stress treatments. The expression pattern observed was specific to a particular genotype indicative of its role in genotype specific regulation in the different geographical climates in which they grow. Furthermore, these genes could be utilized to characterize them and elucidate their specific response mechanisms and ultimate integration into mulberry improvement programs.

## Supplementary Materials

The following are available online at www.mdpi.com/2073-4425/8/9/202/s1. Figure S1: Network of significantly enriched biological process GO terms of ARF genes in *Morus notabilis*. Node size and color intensity represents their significance level. The hypergeometric distribution test with Bonferroni correction was used to assess the significance level. Node size represents the number of GO terms obtained and colour intensity represents their significance level. Table S1: List of primers used in qPCR experiments to estimate the expression level of mulberry ARFs. Table S2: Information about *Morus* ARF members components including CDS and protein length, molecular weight and predicted isoelectric focusing point. Table S3: Significantly Enriched (*p* value ≤ 0.05) Transcription Factor binding sites found in the promoter (URRs) of ARF genes in *Morus*. Table S4: Significant GO terms of the biological process category of ARF genes are shown. Bonferroni corrected *p* values are shown. Table S5: Significant GO terms of the molecular function category for ARF genes are shown. Bonferroni corrected p values are shown. Table S6: Significant GO terms of the cellular component category for ARF genes are shown. Bonferroni corrected *p* values are shown. Table S7: Table showing the FPKM values estimated from RNA-seq.
